# Productivity, niche availability, species richness, and extinction risk: Untangling relationships using individual‐based simulations

**DOI:** 10.1002/ece3.7730

**Published:** 2021-06-16

**Authors:** Euan N. Furness, Russell J. Garwood, Philip D. Mannion, Mark D. Sutton

**Affiliations:** ^1^ Department of Earth Sciences and Engineering Imperial College London London UK; ^2^ Grantham Institute Imperial College London London UK; ^3^ Department of Earth and Environmental Sciences University of Manchester Manchester UK; ^4^ Earth Sciences Department Natural History Museum London UK; ^5^ Department of Earth Sciences University College London London UK

**Keywords:** biodiversity, individual‐based simulation, more‐individuals hypothesis, niche theory, productivity hypothesis

## Abstract

It has often been suggested that the productivity of an ecosystem affects the number of species that it can support. Despite decades of study, the nature, extent, and underlying mechanisms of this relationship are unclear. One suggested mechanism is the “more individuals” hypothesis (MIH). This proposes that productivity controls the number of individuals in the ecosystem, and that more individuals can be divided into a greater number of species before their population size is sufficiently small for each to be at substantial risk of extinction. Here, we test this hypothesis using REvoSim: an individual‐based eco‐evolutionary system that simulates the evolution and speciation of populations over geological time, allowing phenomena occurring over timescales that cannot be easily observed in the real world to be evaluated. The individual‐based nature of this system allows us to remove assumptions about the nature of speciation and extinction that previous models have had to make. Many of the predictions of the MIH are supported in our simulations: Rare species are more likely to undergo extinction than common species, and species richness scales with productivity. However, we also find support for relationships that contradict the predictions of the *strict* MIH: species population size scales with productivity, and species extinction risk is better predicted by relative than absolute species population size, apparently due to increased competition when total community abundance is higher. Furthermore, we show that the scaling of species richness with productivity depends upon the ability of species to partition niche space. Consequently, we suggest that the MIH is applicable only to ecosystems in which niche partitioning has not been halted by species saturation. Some hypotheses regarding patterns of biodiversity implicitly or explicitly overlook niche theory in favor of neutral explanations, as has historically been the case with the MIH. Our simulations demonstrate that niche theory exerts a control on the applicability of the MIH and thus needs to be accounted for in macroecology.

## Graphical abstract

We use simulation experiments to test the predictions of the more‐individuals hypothesis of biodiversity. We find that many of these predictions are supported, but that interspecific competition and limits to niche diversity can provide additional complexity not predicted by the more‐individuals hypothesis.

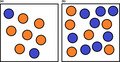

## 1 INTRODUCTION

Species richness in ecosystems is thought to be controlled in some way by productivity, with higher productivity facilitating greater species richness (Allen et al., 2007). This productivity hypothesis has a long history and was originally proposed to explain the latitudinal biodiversity gradient (Hutchinson, 1959), in which species richness declines from low to high latitudes (von Humboldt, 1807). The productivity hypothesis has received consistent but equivocal support (Allen et al., 2007; Brown, 2014; Pianka, 1966; Valentine & Jablonski, 2015; Woolley et al., 2016). Globally, species richness correlates strongly with various measures of environmental energy availability, some of which are reasonable proxies for productivity (Currie, 1991; Hawkins et al., 2003). Indeed, one early articulation of the productivity hypothesis (Wright, 1983) demonstrated its ability to improve global predictions of species richness on islands made by the species–area relationship (MacArthur & Wilson, 1963). Empirical data demonstrate that the productivity hypothesis does not hold in all circumstances. For example, on smaller spatial scales, species richness often peaks at intermediate productivity levels (Mittelbach et al., 2001). Furthermore, some ecosystems confound productivity hypothesis predictions more fundamentally. These include shallow‐water coral reefs, which are more speciose than their productivity would predict, as well as the deep marine realm, which has extremely low productivity but reportedly high species richness (McClain & Schlacher, 2015; Valentine & Jablonski, 2015).

While there is a consensus that a relationship between productivity and species richness does exist in many circumstances, there is no agreement on the precise nature of this relationship (Pontarp & Wiens, 2016; Rabosky & Hurlbert, 2015; Valentine & Jablonski, 2015). Consensus regarding the mechanisms that drive the relationship has also not been reached, although numerous underlying mechanisms have been proposed (Evans et al., 2005). Of these, the “more individuals” hypothesis (MIH) is the simplest and most broadly supported. This proposes that the presence of more individuals in a system is sufficient to promote the coexistence of a greater number of species (Allen et al., 2002; Kisel et al., 2011; Storch et al., 2018). This follows from the assumption that larger species (i.e., those with higher numbers of individuals) will display elevated speciation rates as a consequence of their broader geographic ranges and greater genetic variation (Allen et al., 2007), coupled with an expectation of depressed extinction rates resulting from the reduced impact of random population fluctuations and demographic processes (Pimm et al., 1988). Whereas the former of these assumptions is contested (e.g., Chown & Gaston, 2000), the latter is a cornerstone of multiple, well‐tested ecological “rules,” including the species–area relationship and theory of island biogeography (MacArthur & Wilson, 1963). The MIH predicts that a net gain of species should occur until mean species population size falls to the point at which origination (through speciation and, unless the system is closed, immigration) is in equilibrium with extinction (Allen et al., 2007; Pontarp & Wiens, 2016; Storch et al., 2018). Storch et al. (2018) provided further detailed and formalized predictions of the MIH. These include that: (a) species extinction probability per unit time should decrease with increasing species population size; (b) mean species population size should be independent of total productivity; (c) community abundance (total number of individuals in the system) should be directly proportional to total productivity, and should be reduced by environmental disturbance; and (d) species richness should be directly proportional to community abundance, and should also be reduced by environmental disturbance. Note that although the strict MIH makes no mention of niche diversity (Storch et al., 2018), ecological theory implies that the diversification of species is favored by the presence of unoccupied niches (Brockhurst et al., 2007; Rabosky, 2013; Rabosky & Hurlbert, 2015). Consequently, it is important to ensure that niche diversity does not inhibit the functioning of the MIH when testing the latter.

Testing the MIH has been difficult; experiments that have artificially modified productivity in environments (Armitage, 2015; Asgari & Steiner, 2017; McClain et al., 2018; Tilman, 1993) could only observe community responses on an ecological timescale (except experiments with bacterial communities, e.g., Armitage (2015)). We might expect responses on evolutionary timescales to be different. Furthermore, spatial scale has consistently been identified as a major factor influencing relationships between productivity and species richness (Storch et al., 2018), yet experimental investigation of richness on large scales is not practical. As a result, much of the support for the MIH comes from observational studies of species‐richness gradients (Bonn et al., 2004; Fine, 2015). Such studies are hampered by the fact that many hypothesized drivers of biodiversity covary in space and make similar predictions about observable phenomena (Fine, 2015; Pontarp et al., 2019). These include productivity (Evans et al., 2005), ecosystem stability (Ghalambor et al., 2006), and ambient temperature (Allen et al., 2006). In addition, attempts to verify the MIH (and other productivity–diversity hypothesis mechanisms) have been hindered by disagreement on the precise predictions of these mechanisms (Storch et al., 2018).

Here, we present a series of experiments that evaluate the predictions of the MIH as presented in Storch et al. (2018) within a digital, individual‐based eco‐evolutionary system, REvoSim (Garwood et al., 2019). Simulation studies are becoming increasingly influential in ecology because of their ability to disentangle tightly correlated variables in a way that observational studies cannot (Furness et al., 2021; Pontarp et al., 2019; Pontarp & Wiens, 2016; Saupe et al., 2019; Zurell et al., 2010). However, unlike many previous simulation studies (Gotelli et al., 2009; Münkemüller & Gallien, 2015; van der Plas et al., 2015; Rangel et al., 2018; Ruffley et al., 2019), REvoSim works at the level of the individual, which has the benefit of removing otherwise necessary assumptions about species‐level processes (Pontarp & Wiens, 2016). REvoSim models processes such as mutation, reproduction, and dispersal within a controlled environment and in the absence of ecological interactions more complex than exploitation‐competition. Despite its abstracted nature, REvoSim produces biologically realistic outputs (Garwood et al., 2019) and operates over evolutionary time. Macroecological phenomena, such as species richness gradients, are emergent properties of the model. Although REvoSim lacks certain features (e.g., complex biological interactions), these limitations allow it to be used to test whether such features are required for the generation of real‐world patterns. Here, we apply this approach to testing of the MIH, to studying its interaction with niche availability and, more generally, to the analysis of the relationship between species richness and productivity.

## 2 METHODS

### 2.1 The simulation

Simulations of species dynamics were performed in the Rapid Evolutionary Simulator (REvoSim) version 2.1.0—see Garwood et al. (2019) for a full description of this model and software. REvoSim is an eco‐evolutionary simulator designed to simulate evolution in large populations over geological time. It is open‐source software written in the C++ language supplemented by the QT framework (https://www.qt.io/). It is freely available from https://github.com/palaeoware/revosim, with releases also archived at 10.5281/zenodo.2531610. A full description of the system and software is provided by Garwood et al. (2019); we present an abstracted summary here for convenience.

REvoSim models populations at the level of individual organisms, each of which comprise a 64‐bit binary genome, current energy level, age, and fitness. REvoSim simulations take place within a grid of “cells” (by default 100x100), each of which can contain many organisms (default 100, each in a structure called a “slot”). Cells are characterized by three independent environmental variables, which are visualized as the red, green, and blue color channels of an image. These can be used to provide spatial structure to a simulation and are analogous to variables such as temperature or rainfall in real‐world ecosystems. Environment images can be varied over time in order to provide temporal structure, mimicking disturbance in the real world.

At the start of a simulation, genetically identical organisms are seeded into a single cell in the grid. The simulation then takes place over a number of “iterations” (analogous to years): timesteps during which organisms sequentially: collect energy, attempt to breed and, if they are sufficiently old, die. At regularly spaced “logging iterations” (default every 50 iterations), information about current organisms is output to log files, and the REvoSim speciation algorithm is applied to detect speciation events that have occurred.

### 2.2 Collecting energy

Every iteration, each cell in the simulation is provisioned with a constant amount of energy, determined by the energy level setting of the simulation. This energy is apportioned between all occupants of that cell proportionally to their fitness.

Organism fitness is an integral value between zero and fifteen, determined by an interaction between one half of the organism's genome (the “coding genome”) and the three environmental variables that characterize its cell. At the start of the simulation, three sets of 256 “masks” are generated: one set for each color channel (red, green and blue): one mask for each possible level of that color channel in the environment image. Each mask is 32 bits in length and differs by 1 bit from the masks in the same color channel that represent brightnesses one point brighter or dimmer. For each organism, a “coding bitcount” is calculated as the sum of the number of “1”s (varying between 0 and 96) resulting from an exclusive or (XOR) operation between the coding genome and the red, green, and blue color masks specific to the environment of the cell. The fitness of the organism is then calculated as the settle tolerance parameter (default 15) minus the absolute difference between the fitness target parameter (default 66) and this coding bitcount. Fitness values less than zero are set to zero. This system ensures that there are very many more ways to be unfit within any environment than there are to be optimally fit, but that there are nonetheless a very large number of optimally fit coding genomes.

### 2.3 Breeding

Energy is not lost over time from organisms and accumulates in individuals as they age. Every iteration, after organisms have collected energy, those with more than a user‐defined threshold energy value are entered onto the “breed list” for their cell. Every iteration, each organism on the list will select a partner at random from the populated list to attempt to breed with. If partners are too genetically dissimilar, then this breed attempt fails and no energy is lost by either organism. Otherwise, the organism that initiated the breeding attempt loses a defined amount of energy and an offspring organism is produced. The offspring genome is constructed through random selection from parents for each bit. A single bit of the genome then has a user‐defined defined chance of undergoing a mutation.

Offspring organisms then undergo dispersal. This may result in an individual remaining in the same cell as its parents, or moving to a nearby cell, with probability of dispersal to any given cell being inversely proportional to its distance from the parents’ current position. Once the offspring have moved, each organism attempts to settle, moving into a vacant slot in the cell. This will fail if no vacant slots exist (i.e., if the cell is at capacity) or if the organism's fitness, which is calculated during settling, is zero (i.e., if the organism is very poorly adapted to the environment). If settling fails, then the offspring organism dies.

### 2.4 Species identification

The organisms produced at the start of the simulation are clones and, as such, represent a single species. However, as mutations accumulate, organisms become adapted to different environments and populations form groups that are incapable of breeding with other groups due to genetic dissimilarity. Every logging iteration, REvoSim searches every existing species to determine whether it has undergone speciation. Speciation is identified only if an ancestral species has become partitioned into reproductively isolated clusters: That is, if there are two or more groups of individuals, such that no individual in one group is genetically compatible with any individual in any of the other groups. This algorithm therefore implements the biological species concept (Mayr, 1942). Once speciation has occurred, all members of the less abundant species are assigned a new, unique, species ID number.

### 2.5 Equilibrium

The time required for simulations to reach equilibrium varies depending upon the settings of the simulation and the environment in which organisms are evolving. Equilibria in REvoSim are invariably dynamic: speciation and extinction tend to continue indefinitely but, in the absence of any large changes in simulation conditions, variables such as species richness, mean individual fitness, and total community abundance will fluctuate around equilibrium values after a period of initial directional change.

### 2.6 Comparison with real‐world ecosystems

REvoSim is simplified in a number of ways relative to real ecosystems: It lacks ecological interactions beyond exploitation–competition, and environments have only three axes of variation. However, these simplifications are useful because they mean that any patterns produced by the simulations cannot be dependent on more complex processes such as interference competition and trophic structure, which have been implicated in structuring biodiversity gradients in some hypotheses (Schemske et al., 2009). In the context of the productivity hypothesis, this means that any relationships demonstrated within REvoSim are not the product of the “trophic‐levels” hypothesis (Evans et al., 2005), or related hypotheses that rely upon the number of available niches in the environment being a function of energy level (Evans et al., 2005; McClain et al., 2012).

### 2.7 Evaluated environments

**FIGURE 1:**
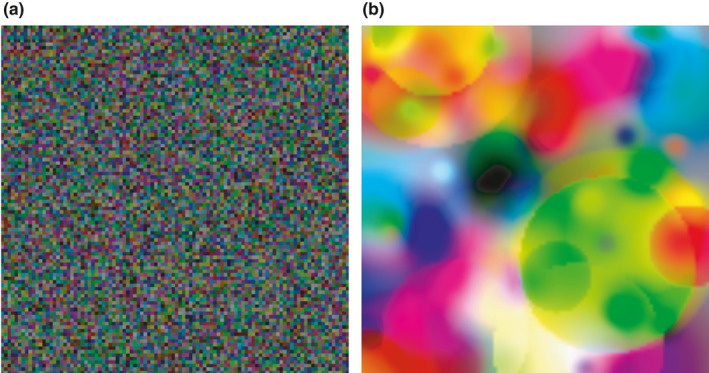
(a) The PS environment. This environment remained static for the duration of the simulations in which it was used. (b) Sample image from the lights environment. Circles in this environment appear, disappear, and move across the space over time

Two different environment types were evaluated: (a) pure spatial heterogeneity (PS) (Figure 1a), in which each pixel's color is random and not correlated with the colors of neighboring pixels, and where pixel colors do not change over time, and (b) “lights” (Figure 1b), an environment consisting of colored circles that move gradually through space, brighten and fade, and intersect. The PS environment was used because it has an easily approximable number of niches (one for each cell in the 100 × 100 grid), a niche here being defined as a unique combination of environmental variables to which a species can specialize. The light environment was used because it produces environmental gradients, temporal structure, and habitat patches of variable size, all of which are present in real environments and none of which are present in the PS environment.

Both PS and lights environments were generated using the EnviroGen software package (Garwood et al., 2019). The PS environment was generated using the “Noise” tool, with the minimum value set to 0 and the maximum value set to 255. The first environment image was taken from the output of this tool and combined with an all‐black image using the “combine” tool, with 50% input from each image, to create the image used. The lights environment was generated using the “dynamic 2” tool with settings modified to increase environmental heterogeneity (Table S2).

### 2.8 Simulation settings

REvoSim settings used in the experiments are recorded in Table S1. Each simulation employed obligate sexual reproduction (rather than asexual, or facultatively sexual reproduction). Organisms had a maximum lifespan of 15 iterations and, where environments were dynamic, linear interpolation of color was used between temporally adjacent images, which were cycled every 100 iterations. Productivity was modified through the “Energy input” parameter in REvoSim, which is functionally equivalent to per cell primary productivity. Python scripts were used to interpret outputs from the model (Code [Link], [Link], [Link], [Link]).

### 2.9 The experiments

In each experiment, sampling occurred once the simulation had reached equilibrium. The number of iterations required for this to occur varies based on simulation settings, but was achieved in all experiments, with the exception noted for part of Experiment 3. A summary of these experiments is provided in Table 1.

**TABLE 1:** A summary of experiments 1–8. “Duration” is the last iteration number at which sampling occurred. “*n*” is the number of independent simulations run in the experiment

Experiment #	Environment	Duration	*n*	Intended to determine the impact of…
1	PS	45,000	116	Energy level and niche availability on species richness
2	PS 2x2	45,000	116	Energy level and niche availability on species richness
3a	PS 4x4	45,000	116	Energy level and niche availability on species richness
3b	PS 4x4	450,000	116	Energy level on time to species richness equilibration
4	Lights	45,000	208	Environmental disturbance and energy level on total community abundance
5	Lights	90,000	5	Species size on species extinction risk
6	Lights	90,000	5	Parent species size on daughter species production rate
7	Lights	90,000	1,552	Total community abundance on mean species size
8	Lights	90,000	1,168	Energy level on community evenness

Experiments 1, 2, and 3 were designed to determine how the shape of the relationship between species richness and productivity changed as the total number of niches in the environment varied. These experiments took place in either the PS environment (Experiment 1) or modified PS environments where the individual areas of color had been expanded from single pixels to either 2 × 2 (Experiment 2) or 4x4 (Experiment 3) pixel areas using a Python script (Code S5 and S6). These modifications changed the number of niches in a predetermined way (i.e., changing color blocks from single pixels to 2 × 2 pixel squares reduced the number of niches by a factor of 4; 4 × 4 pixel squares by a factor of 16). Here, and in later experiments, the number of levels of productivity investigated was somewhat arbitrary, but was selected on the basis that it showed the full range of variation that could be produced by the simulations. 116 simulations were run in each of experiments 1–3, and each simulation had one of 29 levels of productivity. An additional 116 simulations were run in Experiment 3, with species richness measured after 450,000 iterations rather than the default 45,000, in order to ensure that the simulation reached equilibrium. Mean individual fitness (how well‐adapted organisms are to collect energy from their current environment) was also measured in these simulations as an alternate measure of the degree to which species had adapted to environmental heterogeneity.

A further five experiments (4–8) were conducted to investigate the mechanisms responsible for controlling the relationship between species richness and energy level observed in experiments 1–3. These experiments all took place in the lights environment because of its more realistic environmental conditions, and because the relationship between niche availability, which is not well defined in the lights environment, and species richness was clearly defined in experiments 1–3.

Experiment 4 was designed to test the hypothesis that higher levels of environmental disturbance reduce total community abundance at any given energy level (see Storch et al., 2018). 208 simulations were run in this experiment, each with one of 52 possible energy levels and one of two possible environmental refresh rates (environments persisted either indefinitely or for 200 iterations). Indefinite persistence of an environment was achieved by loading in only the first environment image from a set of lights environment images. The number of simulations is higher in this experiment than in experiments 1–3 as there are more possible treatments.

Experiment 5 was designed to test the hypothesis, common to numerous ecological theories including the MIH (Storch et al., 2018), that smaller species (i.e., those with lower numbers of individuals) have higher rates of extinction than larger species. Five simulations were run in Experiment 5. This is much fewer than in experiments 1–4 because each simulation produced multiple data points. In order to ensure that the relationship between species size and extinction rate was not dependent on energy level, each simulation in this experiment had a different energy level. Hybridization between species was prohibited in this experiment because it had a disproportionate impact on the extinction rates of rare species. This prohibition is justified on the grounds that species that survive only through hybridization are not true biological species. In each simulation, every extant species was sampled every 50 iterations between a minimum and maximum iteration (60,000 and 90,000). Sampling consisted of recording species population size and whether the species was still present 500 iterations later. The simulation duration is greater than in experiments 1–4 because the sampling range (60,000–90,000 iterations) is long, and must only include equilibrium dynamics. A wide range of species sizes needed to be examined in order for the impact of species size on extinction rate to be apparent. To ensure that small sample sizes (i.e., low numbers of species of a given size) did not introduce substantial noise into the results, species were grouped into “size classes” for analysis. Each size class had a range of 20 (e.g., species with a population size of 1–20 were grouped into a single size class).

Experiment 6 was designed to test the hypothesis that more abundant species produce daughter species at higher rates than less abundant species. Five simulations were run in Experiment 6, each with one of five energy levels. Every 50 iterations between two bounds (60,000 and 90,000), each species in the simulation was sampled. Sampling consisted of recording the population size of the species, and number of new daughter species in the next iteration. As in Experiment 5, species in Experiment 6 were binned into “size classes,” each with a range of 500, in order to reduce noise in the results that would have been caused by small sample sizes of large species. As in Experiment 5, energy level was varied in Experiment 6 to ensure that the relationship between rate of daughter species production and species size was not dependent on energy level.

Experiment 7 was designed to test the hypothesis that species population size is not a function of total community abundance. 1,552 simulations were run with every species in the 90,000th iteration sampled for population size. Each simulation had one of 97 energy levels. Both the number of simulations and the number of different energy levels sampled were increased in Experiment 7 relative to Experiment 4 because the data contained a greater magnitude of noise and, as such, a larger sample size was needed to confidently detect or reject the presence of trends.

Following the results of Experiments 5 and 6, Experiment 8 was designed to test if mean species evenness (a measure of the disparity in species sizes within an ecosystem) within simulations changed as a function of energy level. This experiment used the same 1552 simulations as Experiment 7. Shannon's evenness (Pielou, 1975) was determined for each iteration between 60,000 and 90,000 in each simulation, and these data were used to calculate the mean evenness for each simulation.

All statistical analyses were performed in R (R Core Team, 2020). Analyses of experiments 1 and 4–8 involved linear models, some of which were “segmented” using the “segmented” package (Muggero, 2008), meaning that breakpoints were fitted into the model at which the coefficient of the relationship between variables changed. Where single models are presented, “*df*” represents the number of degrees of freedom in the model. Where they are compared, “ddf” represents the difference in the number of degrees of freedom between the models. Reported R^2^ values are the adjusted R^2^ values produced in R (R Core Team, 2020).

White et al. (2013) objected to the use of frequentist statistics in simulation studies for two reasons: Firstly, simulation studies can be replicated arbitrarily, until effects are significant even if they are so small as to be biologically meaningless; and secondly, because model parameters are explicitly manipulated between different treatments, the null hypothesis that there is no difference between the treatments is known a priori to be untrue, and will therefore always be rejected given sufficient data. We do not follow these recommendations herein for two reasons: Firstly, there is no difference between the ability of a statistical test to detect a significant effect when it is applied to simulated or nonsimulated data, and the objection of White et al. (2013) applies not specifically to simulated datasets but to large datasets, regardless of source. Use of frequentist statistics on a simulated dataset of limited size is therefore appropriate, and we do not believe that these criticisms apply. Secondly, the suggestion that changes in model inputs will always result in changes in outputs is untrue for models with any degree of stochasticity. A sufficiently complex model could, in principle, be indistinguishable from reality and, as such, this claim is equivalent to suggesting that a change to any one parameter in the real world will predictably change all other measurable parameters. We acknowledge, however, the concerns that White et al. (2013) raise regarding the conflation of statistical significance with biological significance. To ameliorate these, we report effect sizes (in the form of R^2^ values) alongside significance tests and highlight results where effect sizes are statistically significant, but notably small.

## 3 RESULTS

### 3.1 Experiments 1–3: Impact of niche availability on species richness

**FIGURE 2:**
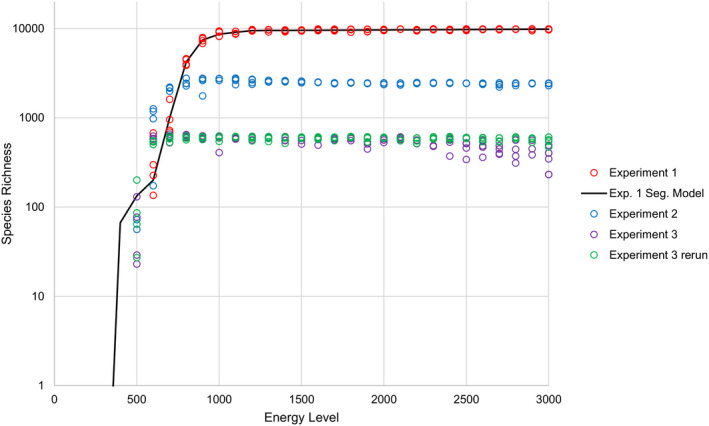
Plot of species richness against energy level in experiments 1–3, under four scenarios with approximately known niche availability (PS, and modified PS environments). The y‐axis is log‐transformed for ease of interpretation. Species richness values of zero are hidden by the log‐transformed axis. At low energy levels (e.g., 500), species richness is zero in environments with many, smaller niches, but greater than zero in environments with fewer, larger niches. The output of the segmented linear model is also plotted, for comparison

**FIGURE 3:**
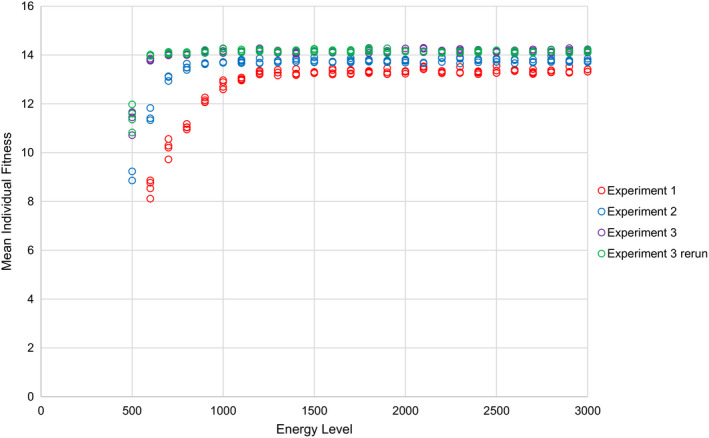
Plot of mean individual fitness of organisms against energy level in experiments 1–3, under four scenarios with variable niche sizes. Unlike species richness, mean individual fitness does not peak at intermediate energy levels in any of the scenarios. Mean individual fitness plateaus more rapidly, and at a higher value, in environments with fewer, larger niches

In Experiment 1, which took place in the PS environment, ANOVA indicates that species richness is predicted well by a linear function of energy level with four segments (*p* < 2.2 × 10^‐16^, *F* = 20,046, *df* = 7, *R* ^2^ = 0.9964) (Figure 2; Table S3). In both the first and last segments (energy level less than 676.826 ± 5.229 and energy level greater than 1,199.998 ± 29.516, respectively), the slope of the relationship between energy level and species richness is low; not significantly different from zero in the first segment (coefficient = 0.66628 ± 0.71872) and marginally greater than zero in the last segment (coefficient = 0.18364 ± 0.103258). In the intermediate segments, species richness has a strong positive relationship with energy level (coefficient = 32.127 ± 1.607 and 4.2118 ± 1.6072, respectively; Table S3). Given that it is clear that species richness saturates above a certain energy level, the slight positive relationship between energy level and species richness in the 4th segment is likely the result of that segment capturing some of the impact of energy level on species richness from before this saturation occurs. The relationship between energy level and mean individual fitness parallels the relationship between energy level and species richness (Figure 3).

In Experiment 2, which took place in a modified PS environment with 25% of the number of niches of Experiment 1, species richness shows a similar relationship to energy level as in Experiment 1 (Figure 2). However, in Experiment 2, species richness becomes saturated at approximately 2,500 species, rather than the 10,000 in Experiment 1. Furthermore, there is a peak in equilibrium species richness at intermediate energy levels in Experiment 2, rather than an increase with energy level up to a point, followed by saturation, as in Experiment 1 (Figure 2). Unlike species richness, mean individual fitness does not display a peak at intermediate energy levels in Experiment 2 (Figure 3). Mean individual fitness saturates at a higher value in Experiment 2 than it does in Experiment 1.

In Experiment 3, which took place in a different modified PS environment from Experiment 2 and which possessed 1/16th of the number of niches of Experiment 1, species richness shows a similar relationship to energy level as in experiments 1 and 2 (Figure 2). However, in Experiment 3, species richness becomes saturated at approximately 625 species, rather than the higher values in experiments 1 and 2. As in Experiment 2, the highest species richnesses in Experiment 3 are found at intermediate energy levels. However, the results of Experiment 3 differ from those of Experiment 2 in that species richnesses at high energy levels in the former fall more notably below the apparent saturation point of approximately 625 species. Similarly to Experiment 2, mean individual fitness in Experiment 3 does not peak at intermediate energy levels (Figure 3). Mean individual fitness saturates at a higher value in Experiment 3 than it does in experiments 1 and 2.

When Experiment 3 was rerun with species richness sampled after 450,000, rather than 45,000, iterations, species richnesses at high energy levels typically no longer fall far below the apparent saturation level (Figure 2). However, the highest species richnesses still occur at intermediate energy levels.

### 3.2 Experiment 4: Impact of disturbance and energy level on total community abundance

**FIGURE 4:**
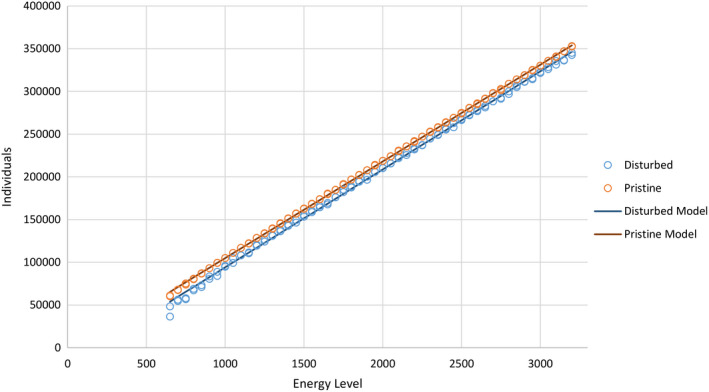
Plot of total community abundance (individuals) after 45,000 iterations against productivity (energy level) in Experiment 4. Total community abundance is a linear function of energy level and is reduced by environmental disturbance. The output of the linear model is also plotted, for comparison

In Experiment 4, which includes environmental disturbance, energy level has a significant positive impact on total community abundance, regardless of the disturbance level (*p* < 2.2 × 10^−16^, *F* = 2.51 × 10^5^). However, environments with lower levels of environmental disturbance have slightly (but consistently) higher community abundances for any given energy level (*p* < 2.2 × 10^‐16^, *F* = 766.93), an effect that is more pronounced at lower energy levels (*p* =.000992, *F* = 11.164; Figure 4) (Table S4). At low energy levels, disturbed environments sometimes display lower total community abundance than a linear relationship would predict. Otherwise, the relationship is linear.

### 3.3 Experiments 5–6: Impact of species size on speciation and extinction

**FIGURE 5:**
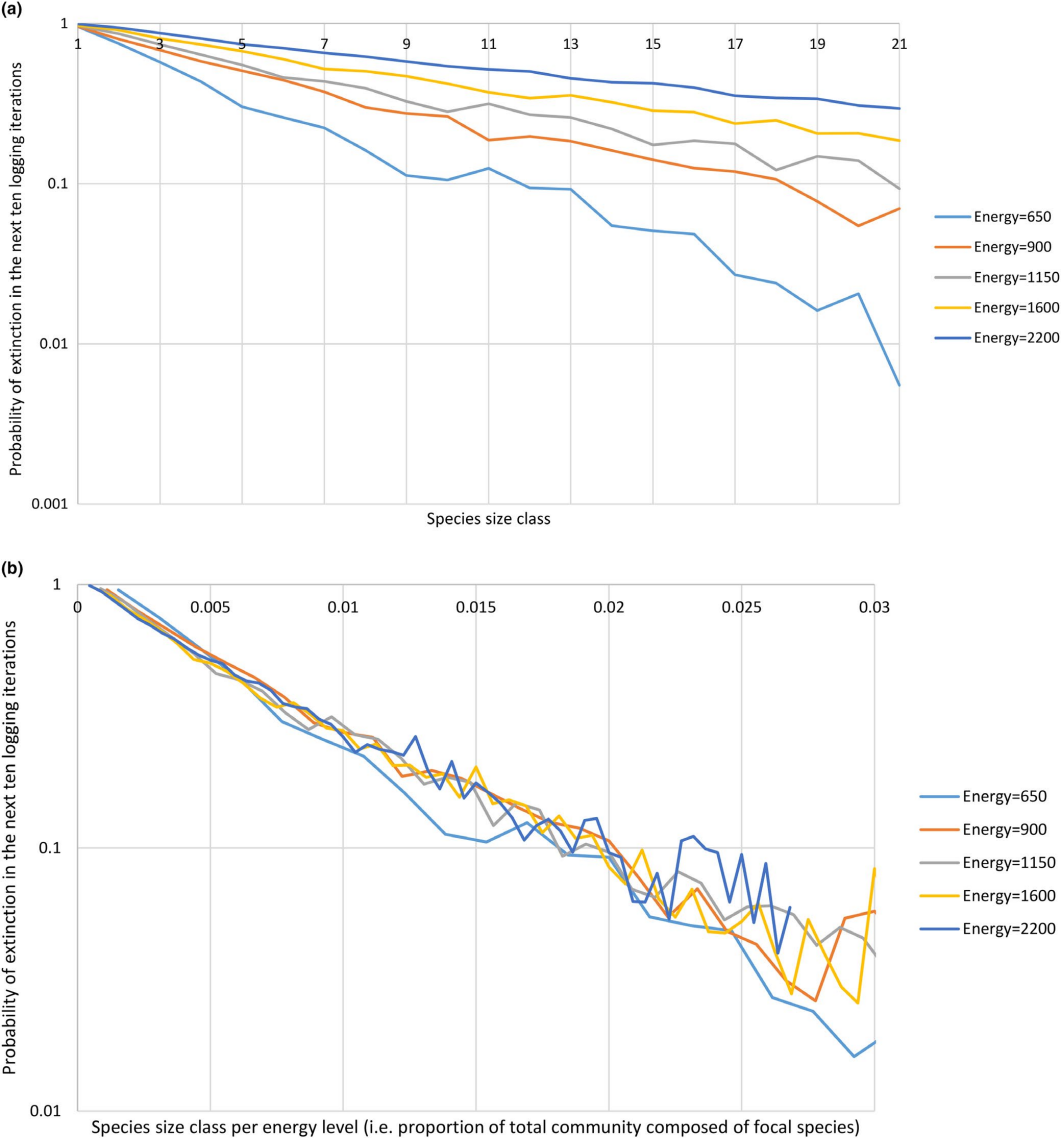
Plot of the probability of a species becoming extinct within 500 iterations in Experiment 5 against (a) species size class or (b) species size class divided by energy level. Individuals are not permitted to hybridize with other species regardless of whether or not the individuals are sufficiently genetically similar. Each point is calculated as a mean of binary outputs (1 = extinction, 0 = no extinction) for every species in every logging iteration (every 50 total model iterations) between 60,000 and 90,000 total model iterations. Each species size class (p) contains all species of size greater than (p–1)*20 and less than p*20 + 1. Larger species are less likely to go extinct, and higher energy level simulations have higher extinction rates for species of any given size. *y*‐axis scaling is log(10). The x‐axis is artificially truncated in both graphs, as small sample sizes produce substantial random noise as size class increases

In Experiment 5, the probability of a species becoming extinct within 500 iterations is negatively related to its population size at time of sampling (*p* < 2.2 × 10^−16^, *F* = 302.56, *R* ^2^ = 0.5428; Table S5; Figure 5a). However, this extinction risk is much better predicted if species population size is first divided by energy level (*p* < 2.2 × 10^−16^, *F* = 2001.3, *R* ^2^ = 0.8873; Tables S6 and S7; Figure 5b). In this case, the model predictor is the proportion of total community abundance accounted for by the focal species. However, even this model has some nonrandom error: A simple log‐linear model (Table S6) underpredicts extinction rates of species with small absolute population sizes. Predictions are significantly improved, specifically for these rare species, by the addition of absolute species size as a variable (Tables S8 and S9).

**FIGURE 6:**
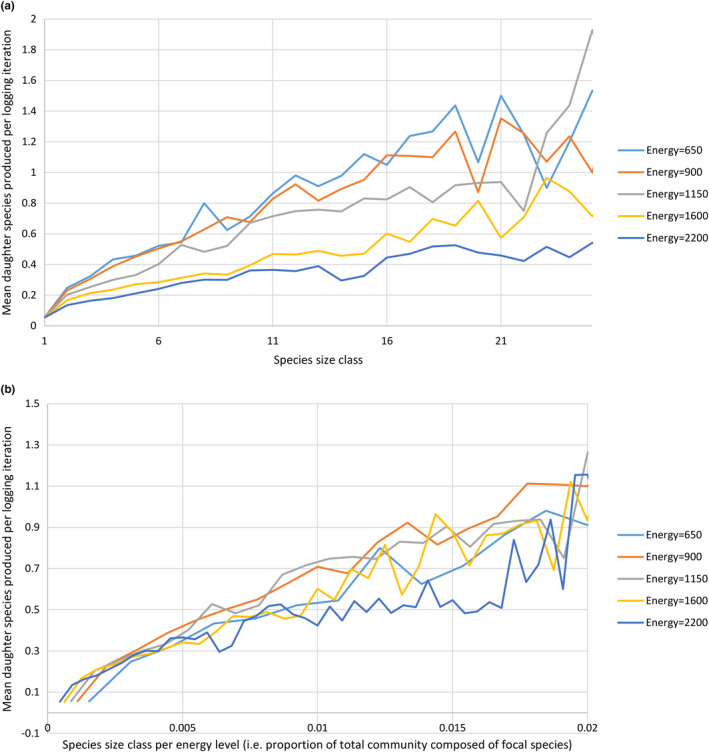
Plot of mean number of daughter species produced by each species of (a) a given size, or (b) a given proportion of the total community abundance, in Experiment 6. Each point is calculated as the mean number of daughter species for every species in every logging iteration (every 50 total model iterations) between 60,000 and 90,000 total model iterations. Each species size class (p) contains all species of size greater than (p–1)*500 and less than p*500 + 1. Species that make up a greater proportion of the total community abundance of the simulation produce more daughter species. The *x*‐axis is artificially truncated in both graphs, as small sample sizes produce substantial random noise as size class increases

In Experiment 6, the rate of daughter species production is positively related to the population size of the parent species at time of sampling (*p* < 2.2 × 10^‐16^, *F* = 132.59, *R* ^2^ = 0.4042; Table S10; Figure 6a). However, as with extinction rate, the rate of daughter species production is much better predicted if species size is first divided by energy level (*p* < 2.2 × 10^−16^, *F* = 345.99, *R* ^2^ = 0.6401; Tables S11 and S12; Figure 6b).

### 3.4 Experiment 7: Impact of energy level on species size

**FIGURE 7:**
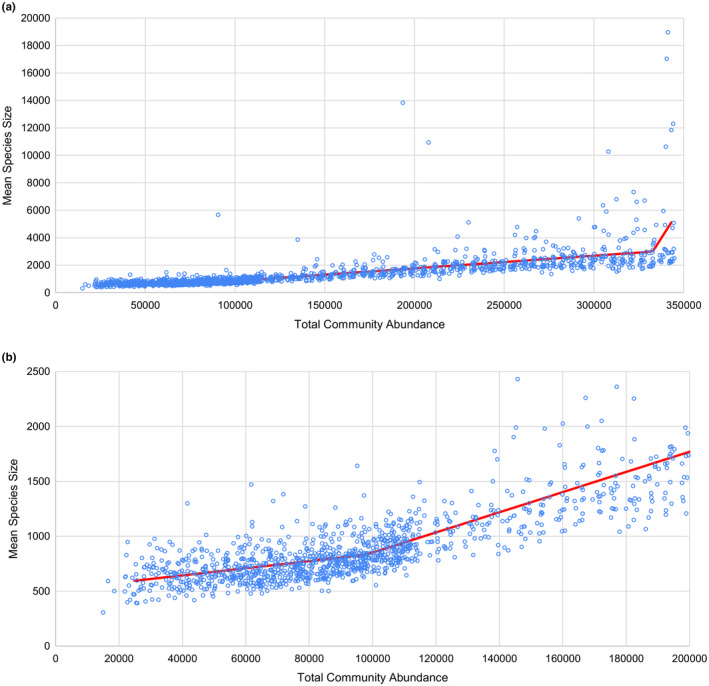
Plots of mean species size against total community abundance in Experiment 7. Each point is calculated as the mean species size of species at 90,000 iterations. Segmented regression indicates that mean species size increases significantly with total community abundance throughout, but that the rate of increase is higher at higher total community abundances. (a) All points, (b) points with total community abundance less than 200,000, and mean species size less than 2,500. This view better displays the increase in the slope of the positive relationship at low total community abundances

In Experiment 7, mean species size can be expressed as a positive linear function of total community abundance (*p* < 2.2 × 10^−16^, *F* = 1,250.6, *df* = 1, *R* ^2^ = 0.4462; Table S13; Figure 7a). However, ANOVA indicates that a linear model with three segments, splitting the graph at total community abundances of 97,978 ± 13,290 and 332,763 ± 1,419, provides a significantly better prediction of mean species size than a single linear regression (*p* < 2.2 × 10^−16^, *F* = 34.401, ddf = 4, *R* ^2^ = 0.4901) (Table S14). In this three‐segment model, total community abundance has a significant positive impact on mean species size in all three segments (Table S15), but segments containing regions of higher total community abundance show a stronger positive impact of total community abundance on mean species size (Figure 7b).

In Experiment 8, evenness is very variable in high energy simulations. High energy simulations take longer to equilibrate (as demonstrated by Experiment 3); this translates, in dynamic environments, into a reduced species richness and potentially misleading evenness score. For this reason, simulations with energy levels greater than 2,000 were excluded from the final analysis in Experiment 8, leaving a sample size of 1,168. In this sample, community evenness could be modeled as a positive linear function of energy level (*p* =.0002637, *F* = 13.395, *R* ^2^ = 0.01052; Table S16). This fit, while significant, is not strong (the model explains a very small proportion of the variance in the data). ANOVA indicates that a linear model with three segments, splitting the graph at energy levels of 624.4 ± 8.3 and 1,019.9 ± 68.8, provides a significantly better fit to the data (*p* = 4.679 × 10,^‐10^ *F* = 12.621, ddf = 4, *R* ^2^ = 0.04849) (Table S17), although this fit is still not strong. In this model, energy level has a significant positive impact on mean community evenness in the first and second segments, and no significant impact in the third segment (Table S18).

## 4 DISCUSSION

### 4.1 Assumptions: Ecological limits theory

In our simulations, variables such as species richness, total community abundance, and mean species size are all measured at equilibrium. In suggesting that these values are useful for making inferences about the real world, we make the implicit assumption that these variables are at, or close to, equilibrium in environments in the real world and, therefore, that the ecological limits theory is accurate.

Ecological limits theory states that real‐world ecosystems tend to be at equilibrium in terms of species richness. This equilibrium reflects a state where species richness has increased to the point that the addition of further species to the ecosystem accelerates extinction by reducing the resources available to them (Rabosky & Hurlbert, 2015). This theory is controversial: Opponents argue that fossil evidence in favor of ecological limits is flawed (Benton & Emerson, 2007), that apparent slowdowns in diversification rate over time are not good evidence of limits to diversity (Moen & Morlon, 2014), and that experimental evidence, including that from biological invasions (Sax et al., 2002), supports the idea that diversity is not, in practice, limited by simple equilibria, but that ecosystems tend to be able to accommodate more species than they naturally contain (Harmon & Harrison, 2015). Nevertheless, proponents of ecological limits argue that it is supported by both paleontological and modern ecological evidence. Paleontological support comes from slowdowns in diversification rate (Cardillo et al., 2005), rebounds in species richness following extinction events (Erwin, 2001), and the relative constancy of species richness through time (Alroy et al., 2008; Benson et al., 2016; Close et al., 2019, 2020). Ecological support comes from correlations between ecological variables and species richness (MacArthur & Wilson, 1963; Wright, 1983), the relative resistance to biological invasion of diverse communities (Tilman, 1999), and the demonstrable explanatory power of other ecological theories that attribute patterns to diversity equilibria (Rabosky & Hurlbert, 2015), such as the widely supported theory of island biogeography (MacArthur & Wilson, 1963; Wright, 1983).

Resolution of this debate is beyond the scope of the present study, although we note here that ecosystems could be influenced by ecological limits without ever reaching them (Cornell, 2013). Nevertheless, the MIH also makes the assumption that ecological limits act in the real world (Storch et al., 2018) and, therefore, by the addition of this assumption we are adding no further conditions to the scenarios in which our results support the MIH.

### 4.2 The shape of the species–energy relationship

Regardless of environment, simulated species richness increases with energy level at first, but this relationship disappears above some threshold energy level. In those experiments in which the number of simulated niches is approximately known (experiments 1–3), species richness saturates when it is approximately equal to the number of available niches (Figure 2). This implies that niche limitation controls species richness above a certain threshold energy level by restricting diversification through ecological speciation (Rabosky, 2013; Rabosky & Hurlbert, 2015). Because organisms have a limited lifespan, repeated failures to breed as a result of genetic incompatibility might result in loss of all reproductive success. In REvoSim, the ability of a pair of organisms to breed is determined by the number of pairwise differences between their genomes (Garwood et al., 2019). Consequently, organisms are selected to have a similar genome to other organisms in the same cell, a process that opposes genetic drift. As such, ecological speciation is likely to be the main process facilitating speciation and, therefore, loss of this process via niche saturation results in a decoupling of species richness from energy level.

The observed initial positive relationship between species richness and energy level supports the productivity hypothesis of species richness. Furthermore, it supports the more‐individuals hypothesis (MIH) as the underlying mechanism (Storch et al., 2018), as the ecological interactions required to test the “trophic‐levels” hypothesis (Evans et al., 2005; Pontarp, 2020) are not present in our simulations. However, it also suggests that there are limits to the MIH: Additional productivity has no positive impact on species richness when the latter is limited by other factors, such as niche availability. Whether or not these other limitations commonly act in nature is unclear (Cornell & Lawton, 1992; Olivares et al., 2018): Evidence suggests that diversification slowdown due to niche saturation does exist (Brockhurst et al., 2007; Ghosh‐Harihar & Price, 2014; Price et al., 2014), although in some cases additional energy can ameliorate this saturation (Ghosh‐Harihar & Price, 2014). Furthermore, the hypothesis that niche saturation reduces diversification rate is at the heart of the theoretical framework of adaptive radiations (Brockhurst et al., 2007; Stroud & Losos, 2016). However, other workers have argued that measurements of diversification slowdown are problematic (Harmon & Harrison, 2015) and that niche availability does not constrain speciation in practice (Harmon & Harrison, 2015; Jetz & Fine, 2012; Ricklefs & Bermingham, 2001; Wiens, 2011).

While we can confidently rule out any influence of trophic levels in REvoSim, we cannot immediately rule out the influence of “evolvability”; that is, that higher productivity simulations display higher species richness not because of differences in species extinction risk but because a larger number of individuals leads to a greater number of mutations in the population as a whole, increasing the opportunities for adaptive evolution to occur (Olson‐Manning et al., 2012). However, our results suggest that this mechanism is not the driving force behind our observed patterns: higher energy levels led to relatively low speciation rates in Experiment 6 (Figure 6a), and this pattern persisted even if species population size was measured relative to the total community abundance rather than in absolute terms (Figure 6b).

Apparent peaks in species richness at intermediate energy levels in more structured environments (i.e., experiments 2 and 3, with modified pure spatial heterogeneity environments) almost certainly reflect the simulation speciation system. REvoSim's species identification algorithm identifies new species through an exhaustive search of genomes within existing species. It then partitions daughter species from parent species if a group of individuals can be identified where no member of that group is genetically able to breed with any member of the parent species outside of that group. One consequence of this is that when species have larger populations (and, therefore, greater genetic variability), it is less likely that a daughter species will be identified, because strict genetic isolation is harder to achieve. This artifact explains the following two phenomena. Firstly, species richness exceeds environmental niche diversity at intermediate energy levels because ephemeral species (Rosenblum et al., 2012) are more likely to be labelled as new species by REvoSim when they are daughters of low‐population species. This can be observed in both experiments 2 and 3 (Figure 2), where species richness exceeds the hypothetical niche richness of 2,500 and 625, respectively, at intermediate energy levels. Notably, this does not occur in Experiment 1, presumably because niches in Experiment 1 consist of single cells and, therefore, multiple species cannot coexist for an extended period of time within the same niche due to the substantial risks of breeding failure. Secondly, species richness takes longer to reach equilibrium at higher energy levels due to the increased time required for daughter species of larger species to be detected. This can be observed in Experiment 3 (Figure 2), where allowing longer for equilibration to occur largely removes cases of high energy level simulations with species richnesses substantially below the hypothetical niche diversity of 625. These interpretations are supported by the observation that mean individual fitness does not display peaks at intermediate energy levels, or reduced rates of equilibration at high energy levels, which implies that neither the intermediate energy level peaks, nor the high energy level drop‐offs in species richnesses, are informative about the degree to which organisms are adapted to their environment in experiments 2 and 3 (Figures 2 and 3). This implies that the effects of energy on species richness, excepting the initial positive relationship and subsequent plateau, are not real, biological effects. Rather, they are artifacts resulting from the inherent difficulty in detecting incipient species in populations of individuals (Rosenblum et al., 2012). However, this difficulty is not unique to REvoSim; similar difficulties exist in the real world (Mallet, 2008), and so while these effects might reasonably be considered artifacts, that does not imply that they have no relevance to the interpretation of empirical data.

In those environments in which the number of niches is approximately known (i.e., experiments 1–3), there is a clear, positive relationship between the number of niches in the environment and the energy level required to reach saturation in both species richness and mean fitness (Figures 2 and 3). This implies that species richness should correlate strongly with energy level over a wider range of productivity values in an environment with a higher diversity of niches, whereas species richness should saturate quickly as productivity increases in a niche‐poor environment. In the real world, all species occupy niche space, but they can also create it (Jones et al., 1994). Furthermore, the fitness landscape is substantially more complex in the real world than in our simulations. As such, it might be possible for species richness to scale with energy in the real world over a large range of energy levels, as niche diversity can be expected to increase as the number of species increases.

An additional effect of niche diversity is observed at the lowest energy levels. Under these circumstances, species richness is inversely proportional to niche diversity (i.e., simulations with fewer, larger niches can support more species at lower energy levels than simulations with more, smaller niches: Figure 2). This could be because simulations with more niches result in a higher probability of organisms either failing to find a compatible mate (due to greater immigration of genetically incompatible organisms from neighboring niches) or failing to establish after initial dispersal (due to a higher probability of dispersing into a niche to which they are not adapted), that is, an “edge effects” phenomenon (Razafindratsima et al., 2017). This is a biologically realistic phenomenon: It is well established that small habitats can result in extirpation of species due to their loss of recruits by dispersal out of the habitat (Haddad et al., 2015), and that highly heterogeneous habitats can reduce species richness by failing to provide sufficient resources for specialists to survive (Allouche et al., 2012). This hypothesis is supported by the observation that mean individual fitness plateaus at a higher value in environments with fewer, larger niches than in environments with many, smaller niches (Figure 3).

### 4.3 Prediction verification—the more‐individuals hypothesis

**FIGURE 8:**
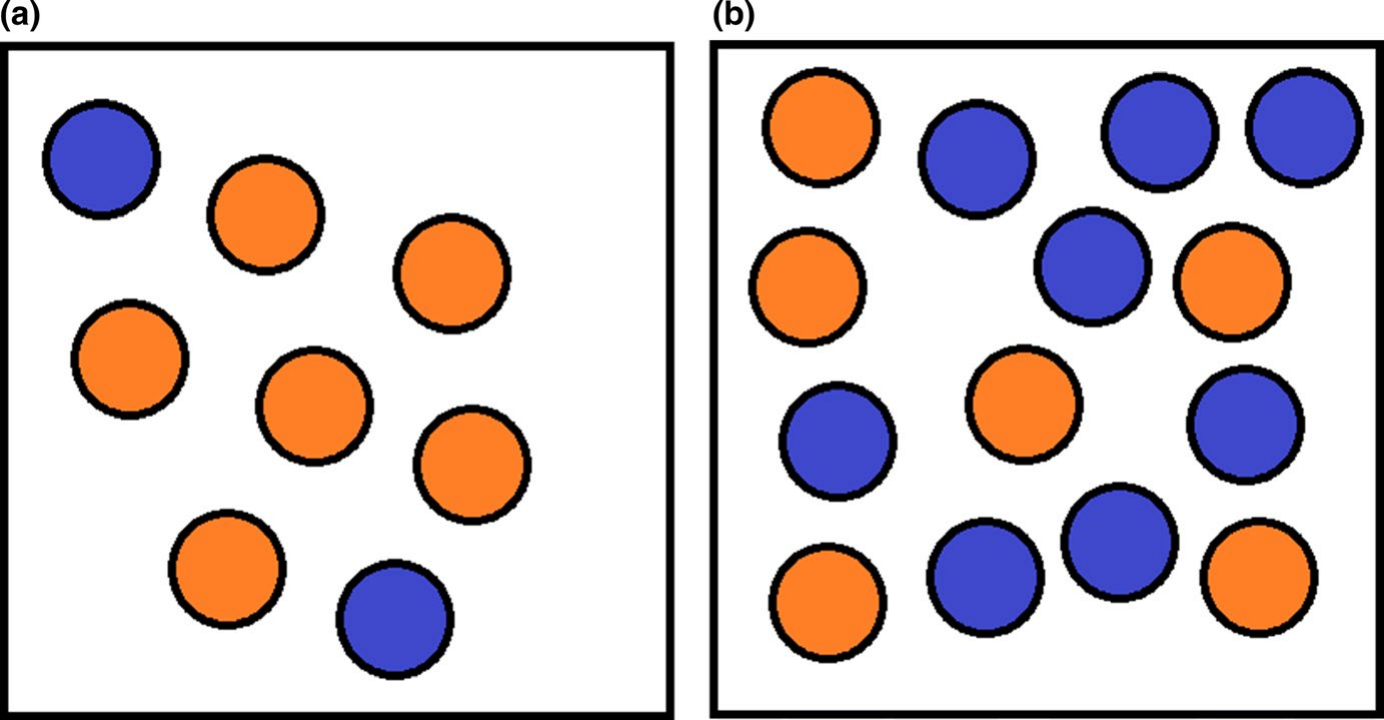
Illustration of the conditions experienced by a focal species of a constant size (orange) under conditions of (a) relatively low energy or (b) relatively high energy. When the energy level is relatively low, the focal species encounters a lower proportion of individuals from other species (blue) with which it cannot breed. As such, it is more likely to succeed in breeding attempts when paired with a random individual in its cell

The MIH, as formalized in Storch et al. (2018), attributes the relationship between species richness and energy level to the impact of species size on extinction rate. Our experiments recover a clear link between species size and extinction rate: Species with small population sizes are more likely than species with large population sizes to become extinct within a given period of time. However, contrary to the predictions of the MIH, absolute species population size is a worse predictor of extinction risk than relative species population size (i.e., species population size as a proportion of total community abundance) (compare Figure 5a and b). We hypothesize that this is the result of a combination of disruption of breeding in rarer species and ecological drift. The higher the energy level, the more nonconspecific individuals will be present in the environment alongside a focal species of any given size. As such, individuals of the focal species face a greater risk of trying, and failing, to breed with members of another species, unless the focal species is large enough to monopolize a cell. There is also a heightened risk of extinction by ecological drift, which can drive one of two ecologically equivalent species to extinction due to random fluctuations in population size (Svensson et al., 2018; Figure 8).

Both of these mechanisms have real‐world analogues. While a greater risk of failing to breed is a product of the REvoSim breeding mechanism, it can occur in certain scenarios in nature (e.g., species‐blind “mating” attempts mediated by pollen transfer by a generalist pollinator, or production of nonviable hybrid offspring by matings between a focal species and a related species). A greater risk of competitive exclusion by an ecologically equivalent species is the mechanism of species extinction described in the Neutral Theory of Biodiversity (NTB) (Hubbell, 2001). Although the importance of ecology is clear in our simulations generally and, therefore, the NTB is of limited applicability herein, it applies locally within our simulations when multiple species are equally well adapted to the conditions within a single cell. While studies have suggested that the NTB is generally considered to be oversimplified (e.g., Chave, 2004; McGill, 2003; Rapacciuolo & Blois, 2019), it does make accurate predictions about certain aspects of community ecology (Chave, 2004; Li & Ma, 2020). Similar mechanisms could explain the equivalent relationship in rate of daughter species production (Figure 6). In lower‐energy environments, a focal species of a given size will tend to be spread over a greater number of cells (as each cell has a lower carrying capacity); as such, it will have more opportunity to become geographically fragmented and locally adapted, leading to ecological speciation. Furthermore, daughter species can only be detected in our simulations if they survive until the next logging iteration. As such, mechanisms that reduce the extinction rates of new species will also increase the apparent production rate of daughter species. Storch et al. (2018) also predicted that the rate of daughter species production by existing species should be either unrelated to, or a positive function of, species population size. The latter is clearly the case in our simulations: more abundant species speciate more (Figure 6).

Increased extinction rates in species of a given size in higher energy ecosystems should, all else being equal, manifest in the real world as higher mean species sizes in higher energy ecosystems. There is corroborating real‐world evidence for this pattern (Kaspari et al., 2000; Stoner et al., 2018), although the studied ecosystems must be at an eco‐evolutionary equilibrium (Rabosky & Hurlbert, 2015). Regardless of whether population size is measured in absolute terms or as a proportion of total community abundance, extinction risk in our simulations is a negative declining function of population size, as predicted by ecological theory (Evans et al., 2005).

The key prediction of the strictest formulation of the MIH is that mean species size should not vary as a function of total community abundance (Storch et al., 2018). Our results do not support this prediction: Although the slope of the relationship between mean species and total community abundance is not constant, it is always significantly positive (Figure 7). Our results divide the relationship between mean species size and total community abundance into three segments: one at low community abundances, one at intermediate community abundances, and one at high community abundances. Fundamentally, increases in total community abundance must result in some combination of increases in species richness and/or mean species size. At low community abundances, species richness increases as a function of energy level (Figure 2), but so does mean species size (Figure 7). At intermediate community abundances, species richness no longer increases as a function of total community abundance (Figure 2); as such, mean species size must increase more rapidly to compensate (Figure 7). At high community abundances, some simulations have anomalously high mean species sizes. This is likely an artifact produced by the species concept used by REvoSim, which takes more iterations to identify distinct populations as sister species if those populations are larger (hence the difference between standard duration and long duration 4x4 simulations in Figure 2). Because the environment in this experiment is dynamic, new populations of potential sister species are constantly being produced and, as such, simulations with high total community abundances will always have some large species in them that the algorithm has been so far unable to split. The segmentation analysis responds to this by suggesting a significant increase in the rate at which mean species size increases with total community abundance at high total community abundances, although this might not be biologically meaningful.

At equilibrium, species extinction rates must equal species origination rates (assuming a closed system, as in our experiments). The MIH states that increases in total community abundance should allow for division of individuals between a greater number of species without an increase in per‐lineage extinction rates (Storch et al., 2018). However, our experiments suggest that, for species of any given size, per lineage extinction rate will be higher in more populous communities (Figure 5a). If both log(extinction probability) and daughter species production rate were perfectly linear functions of the proportion of total community abundance accounted for by the focal species, then equilibrium species richness would be independent of total community richness, and increases in total community richness could be compensated for only by equivalent increases in mean species size (Equations S1). Clearly, this is not the case, as increases in total community abundance (following increases in energy level) lead to an increase in species richness (Figure 2). This discrepancy can be explained by two factors. Firstly, log(extinction probability) is not a perfectly linear function of the proportion of total community abundance accounted for by the focal species (Table S9): Absolute species population size also contributes to reducing extinction risk. Secondly, it is not only mean species size but also the distribution of species sizes within a simulation that controls mean extinction rate. Because it is the logarithm of extinction risk, rather than simply extinction risk, that is a function of species population size, small decreases in the populations of large species will have very little impact on their extinction risk, whereas an increase in population size of the same absolute magnitude can greatly reduce the extinction risk of a rare species. As such, mean extinction risk can be expected to be reduced, regardless of mean species size, in simulations with higher community evenness (i.e., more equitable distribution of individuals between species). In our simulations, community evenness is higher in simulations with higher energy levels, at least over the range of energy levels where species richness is a function of energy level. The magnitude of this effect is quite small and, as such, it is unlikely that it is the sole driver of differences in species richness in our results. Nevertheless, higher energy level simulations contained more even community abundances and, as such, higher energy level simulations should have lower per‐lineage extinction rates at any given mean species size and, consequently, higher equilibrium species richnesses.

Species richness ceases to scale with energy level above a certain threshold in all of our simulations. This implies that, beyond a certain energy level, some other factor limits species richness. It is clear from the simulations in which maximum niche diversity is approximately known (i.e., experiments 1–3) that this factor is availability of unoccupied niches, which facilitate diversification through ecological speciation (Rundle & Nosil, 2005), at least to the degree allowed by disturbance in the environment (Grant et al., 2004). Our simulations predict that, in real‐world environments where niche space is saturated, increasing productivity should not produce an increase in species richness. However, this is a challenging prediction to test using real‐world data; it is difficult to determine the level of niche occupancy in an ecosystem, although many studies have attempted to do so (e.g., McClain et al., 2018; Pellissier et al., 2018; Price et al., 2014). It is even more difficult to do this in multiple areas that are ecologically similar, but that exist along the gradient of energy required to test the explanatory power of the productivity hypothesis. Furthermore, there are mechanisms that we have not tested in our simulations, such as the trophic‐levels hypothesis (Evans et al., 2005), which could operate at energy levels beyond those at which we have observed the MIH to operate. Nonetheless, there is evidence from the real world that increasing energy levels can result in more densely packed niche space (McClain et al., 2018; Pellissier et al., 2018), and that the latter can become saturated once species richness grows to a sufficient level (Price et al., 2014), resulting in species richness no longer increasing as a function of energy level (Carnicer et al., 2007), as found in our simulations.

## 5 CONCLUSION

Our work finds support for certain core aspects of the productivity hypothesis: Extinction rates are higher in less abundant species, and daughter species production is higher in more abundant species. As the more‐individuals hypothesis predicts, this results in higher species richness in environments with greater total community abundance. However, increases in total community abundance are also accompanied by increases in mean species size that compensate for the increased risks of breeding failure and increased pressures from ecological drift. This increase in mean species size significantly dampens the positive effects of total community abundance on species richness. Furthermore, changes in community evenness can cause changes in species richness independent of the effects of total community abundance, and limits to niche diversity can cap the species richness produced by the mechanisms of the more‐individuals hypothesis. Our results highlight the importance of niche theory in controlling species richness, even with respect to mechanisms such as the more‐individuals hypothesis, which are not intuitively dependent on ecological differences between species. Finally, these experiments provide further evidence that the REvoSim eco‐evolutionary system can reproduce real‐world patterns, and can be used to test specific evolutionary hypotheses.

## CONFLICT OF INTEREST

The authors declare no conflict of interest.

## AUTHOR CONTRIBUTIONS

**Euan N. Furness:** Conceptualization (equal); Data curation (equal); Formal analysis (equal); Investigation (equal); Methodology (equal); Software (supporting); Writing‐original draft (lead); Writing‐review & editing (equal). **Russell J. Garwood:** Software (lead); Writing‐review & editing (equal). **Philip D. Mannion:** Funding acquisition (equal); Project administration (equal); Supervision (equal); Writing‐review & editing (equal). **Mark D. Sutton:** Funding acquisition (equal); Project administration (equal); Resources (equal); Software (lead); Supervision (equal); Writing‐review & editing (equal).

## Supporting information

Tables S1–S18

Code S1

Code S2

Code S3

Code S4

Code S5

Code S6

## Data Availability

REvoSim code is available from GitHub: https://github.com/palaeoware/revosim. Data from experiments is available on the Dryad digital repository: https://doi.org/10.5061/dryad.4tmpg4f9m. Python scripts used for the extraction of species richness, total community abundance, and mean species evenness data are stored in Supplementary code 1 and 2. Scripts used to calculate extinction rates are stored in Supplementary code 3. Scripts used to calculate origination rates are stored in Supplementary code 4. Scripts used to modify environment files are stored in Supplementary code 5 and 6.
