# Supplementary material for: Productivity, niche availability, species richness, and extinction risk: Untangling relationships using individual‐based simulations
Source: Ecol Evol. 2021 Jun 16;11(13):8923–40. doi: 10.1002/ece3.7730 (PMC8258231; doi:10.1002/ece3.7730)
Supplement: Supplementary file 1 — Tables S1–S18 [file ECE3-11-8923-s007.docx]

**Supplementary Information:**

**Supplementary Tables**

Table S1. REvoSim settings used to run simulations.

| **Variable** | **Setting** |
| --- | --- |
| Chance of mutation | 10 |
| Start age | 15 |
| Breed threshold | 500 |
| Breed cost | 500 |
| Max difference to breed | 3 |
| Use max diff to breed | Yes |
| Breed only within species | No (except in experiment 5, where Yes) |
| Breed Mode | Obligate sexual |
| Dispersal | 15 |
| Nonspatial setting | No |
| Environment refresh rate | 100 |
| Environment mode | Bounce |
| Interpolate between images | Yes |
| Toroidal environment | No |
| Grid X | 100 |
| Grid Y | 100 |
| Slots | 100 |
| Fitness target | 66 |
| Energy Input | Variable: see experiments |
| Settle tolerance | 15 |
| Recalculate fitness | No |
| Phylogeny settings | Basic |
| Refresh/polling rate | 50 |
| Logging: Population/Environment | None |
| Logging: To Text File(s) | Write Log Files |
| Exclude species without descendants | No |
| Minimum species size | 0 |
| Don’t update GUI on refresh/poll | No |

Table S2. EnviroGen settings used to generate the lights environment used in the simulations. These settings were entered into the “Dynamic 2” tab.

| **Variable** | **Setting** |
| --- | --- |
| Object Count | 80 |
| Maximum Object Size | 40 |
| Minimum Object Size | 10 |
| Maximum Tightness | 8.00 |
| Minimum Tightness | 0.10 |
| Maximum Velocity | 3.00 |
| Maximum Size Velocity | 1.00 |
| Maximum Colour Velocity | 8.00 |
| Maximum Tightness Velocity | 0.40 |
| Maximum Acceleration | 1.00 |
| Max Size Acceleration | 1.00 |
| Max Tightness Acceleration | 0.10 |
| Max Colour Acceleration | 2.00 |
| Speed Factor | 1.00 |
| Apply accelerations every ... | 1 |

Table S3. ANOVA of a four-segment model of species richness against energy level in experiment 1. Slope values are determined using the “slope” function in the “segmented” package (Muggeo 2008). Adjusted R^2^=0.9964. Reported p values refer to the significance of the variable as a whole, including interaction terms, as opposed to independent of those terms.

| **Variable** | **Slope** | **Upper Slope 95% Confidence Interval** | **Lower Slope 95% Confidence Interval** | **F** | **p** |
| --- | --- | --- | --- | --- | --- |
| Energy Level | 0.66628 | 1.3850 | -0.052455 | 20046.3634 | <2.2x10^-16^ |
| Energy Level * Segment 1 | 32.127 | 33.7340 | 30.520000 | 4233.2205 | <2.2x10^-16^ |
| Energy Level * Segment 2 | 4.21180 | 5.8189 | 2.604600 | 7581.3666 | <2.2x10^-16^ |
| Energy Level * Segment 3 | 0.18364 | 0.2869 | 0.080382 | 89.3237 | 8.258x10^-16^ |

Table S4. ANOVA of the impacts of energy level and pristine environment (environmental refresh rate = 0, as opposed to the default of 200) on total community abundance in experiment 4. Reported p values refer to the significance of the variable as a whole, including interaction terms, as opposed to independent of those terms.

| **Variable** | **Coefficient** | **F** | **p** |
| --- | --- | --- | --- |
| Intercept | -19720 | - | - |
| Energy Level | 114.7 | 251440 | <2.2x10^-16^ |
| Pristine | 1237 | 766.928 | <2.2x10^-16^ |
| Energy Level * Pristine | -1.519 | 11.164 | 0.000992 |

Table S5. ANOVA of the impact of absolute species size class (of a focal species) and energy level on the logarithm (base 10) of the probability of a focal species becoming extinct in the next 500 iterations, where hybridization between species is forbidden.

| **Variable** | **Coefficient** | **F** | **p** |
| --- | --- | --- | --- |
| Intercept | -0.327418 | - | - |
| Species Size Class | -0.025490 | 302.56 | <2.2x10^-16^ |

Table S6. ANOVA of the impact of species size class divided by simulation energy level (i.e. proportion of total community abundance accounted for by focal species) on the logarithm (base 10) of the probability of a focal species becoming extinct in the next 500 iterations, where hybridization between species is forbidden.

| **Variable** | **Coefficient** | **F** | **p** |
| --- | --- | --- | --- |
| Intercept | -0.20808 | - | - |
| Species Size Class / Energy Level | -37.51251 | 2001.3 | <2.2x10^-16^ |

Table S7. ANOVA comparison of the relative explanatory power of absolute species size class and species size class divided by energy level in predicting extinction risk in Experiment 5.

| **Model** | **Residual Sum Squares** | **Residual df** | **R^2^** |
| --- | --- | --- | --- |
| Species Size Class | 37.783 | 253 | 0.5428 |
| Species Size Class / Energy Level | 9.311 | 253 | 0.8873 |

Table S8. ANOVA of the impact of absolute species size class, and species size class divided by simulation energy level (i.e. proportion of total community abundance accounted for by focal species) on the logarithm (base 10) of the probability of a focal species becoming extinct in the next 500 iterations, where hybridization between species is forbidden. Reported p values refer to the significance of the variable as a whole, including interaction terms, as opposed to independent of those terms.

| **Variable** | **Coefficient** | **F** | **p** |
| --- | --- | --- | --- |
| Intercept | 0.059981 | - | - |
| Species Size Class | -0.010898 | 0.2689 | <2.2x10^-16^ |
| Species Size Class / Energy Level | -57.120162 | 3587.7806 | <2.2x10^-16^ |
| Interaction | 0.586152 | 202.2831 | <2.2x10^-16^ |

Table S9. ANOVA comparison of the relative explanatory power of species size class divided by energy level, versus absolute species size class and species size class divided by energy level in predicting extinction risk in Experiment 5.

| **Model** | **Residual Sum Squares** | **Residual df** | **Significance of fit improvement** |
| --- | --- | --- | --- |
| Species Size Class / Energy Level | 9.3112 | 253 | - |
| Species Size Class * Species Size Class / Energy Level | 5.1529 | 251 | <2.2x10^-16^ |

Table S10. ANOVA of the impact of species size class (of a focal species) and energy level on the mean number of daughter species produced in the next 50 iterations.

| **Variable** | **Coefficient** | **F** | **p** |
| --- | --- | --- | --- |
| Intercept | 0.178552 | - | - |
| Species Size Class | 0.036286 | 132.59 | <2.2x10^-16^ |

Table S11. ANOVA of the impact of species size class divided by simulation energy level (i.e. proportion of total community abundance accounted for by focal species) on the mean number of daughter species produced in the next 50 iterations.

| **Variable** | **Coefficient** | **F** | **p** |
| --- | --- | --- | --- |
| Intercept | 0.04868 | - | - |
| Species Size Class / Energy Level | 53.05592 | 345.99 | <2.2x10^-16^ |

Table S12. ANOVA comparison of the relative explanatory power of species size class and species size class divided by energy level in predicting rate of daughter species production in experiment 6.

| **Model** | **Residual Sum Squares** | **Residual df** | **R^2^** |
| --- | --- | --- | --- |
| Species Size Class | 56.772 | 193 | 0.4042 |
| Species Size Class / Energy Level | 34.295 | 193 | 0.6401 |

Table S13. ANOVA of a linear model of mean species size as predicted by total community abundance. Adjusted R^2^=0.4462

| **Variable** | **Coefficient** | **F** | **p** |
| --- | --- | --- | --- |
| Intercept | 54.57 | - | - |
| Total Community Abundance | 0.009142 | 1250.6 | <2.2x10^-16^ |

Table S14. ANOVA comparison of segmented (three-segment) and unsegmented linear models of mean species size as predicted by total community abundance.

| **Model** | **Residual Sum Squares** | **Residual df** | **Significance of Fit Improvement** |
| --- | --- | --- | --- |
| Unsegmented | 1313429909 | 1550 | - |
| Three-Segment | 1206080392 | 1546 | <2.2x10^-16^ |

Table S15. ANOVA of a three-segment model of mean species size against total community abundance in experiment 7. Slope values are determined using the “slope” function in the “segmented” package (Muggeo 2008). Adjusted R^2^=0.4901. Reported p values refer to the significance of the variable as a whole, including interaction terms, as opposed to independent of those terms.

| **Variable** | **Slope** | **Upper Slope 95% Confidence Interval** | **Lower Slope 95% Confidence Interval** | **F** | **p** |
| --- | --- | --- | --- | --- | --- |
| Total Community Abundance | 0.0032568 | 0.0064315 | 0.00008207 | 1358.3918 | <2.2x10^-16^ |
| Total Community Abundance * Segment 1 | 0.0091831 | 0.0099981 | 0.00836800 | 32.5336 | 1.401x10^-8^ |
| Total Community Abundance * Segment 2 | 0.2047000 | 0.2727600 | 0.13663000 | 105.0711 | <2.2x10^-16^ |

Table S16. ANOVA of a linear model of mean community evenness (Shannon’s evenness) as predicted by energy level. Adjusted R^2^=0.0.01052

| **Variable** | **Coefficient** | **F** | **p** |
| --- | --- | --- | --- |
| Intercept | 0.6714 | - | - |
| Energy Level | 1.864x10^-5^ | 13.395 | 2.637x10^-4^ |

Table S17. ANOVA comparison of segmented (three-segment) and unsegmented linear models of mean community evenness (Shannon’s evenness) as predicted by energy level.

| **Model** | **Residual Sum Squares** | **Residual df** | **Significance of Fit Improvement** |
| --- | --- | --- | --- |
| Unsegmented | 4.5113 | 1165 | - |
| Three-Segment | 4.3233 | 1161 | 4.679x10^-10^ |

Table S18. ANOVA of a three-segment model of mean community abundance (Shannon’s evenness) against energy level in experiment 8. Slope values are determined using the “slope” function in the “segmented” package (Muggeo 2008). Adjusted R^2^=0.04849. Reported p values refer to the significance of the variable as a whole, including interaction terms, as opposed to independent of those terms.

| **Variable** | **Slope** | **Upper Slope 95% Confidence Interval** | **Lower Slope 95% Confidence Interval** | **F** | **p** |
| --- | --- | --- | --- | --- | --- |
| Energy Level | 0.0023071 | 0.0044236 | 0.00019062 | 13.929 | 0.000199 |
| Energy Level * Segment 1 | 7.919x10^-5^ | 0.00012019 | 3.8191x10^-5^ | 30.187 | 4.819x10^-8^ |
| Energy Level * Segment 2 | -1.3165x10^-5^ | 4.2489x10^-6^ | -3.058x10^-5^ | 20.298 | 7.295x10^-6^ |

**Supplementary Equations**

Equations S1. Equations describing the relationship between total community abundance and species richness when both log(extinction probability) and daughter species production rate are linear functions of the proportion of total community abundance accounted for by the focal species. E is lineage extinction probability, O is lineage origination rate, I_n_ is the number of individuals in a species n, I_T_ is the total community abundance, and a and b are constants.

$$(1) log(E_{n})=a\frac{I_{n}}{I_{T}}$$

$$(2) O_{n}=b\frac{I_{n}}{I_{T}}$$

$$(3) E_{n}={10}^{a\frac{I_{n}}{I_{T}}}$$

At equilibrium, the sum of all origination rates must equal the sum of all extinction probabilities.

$$(4) \sum_{n=1}^{S} E_{n}=\sum_{n=1}^{S} O_{n}$$

A doubling of total community richness I_T_, not accompanied by any increase in species richness S or change in species evenness, would result in a doubling of species sizes I_n_. From equations 2 and 3:

$$(5) O_{n(new)}=b\frac{2I_{n}}{2I_{T}}=b\frac{I_{n}}{I_{T}}$$

$$(6) E_{n(new)}={10}^{a\frac{2I_{n}}{2I_{T}}}={10}^{a\frac{I_{n}}{I_{T}}}$$

This change does not have any effect on extinction or origination rates. As such, it does not change equilibrium species richness.
